# Retinoschisis associated with Kearns-Sayre syndrome

**DOI:** 10.1080/13816810.2020.1799416

**Published:** 2020-08-13

**Authors:** Julia Chertkof, Robert B. Hufnagel, Delphine Blain, Andrea L. Gropman, Brian P. Brooks

**Affiliations:** a Ophthalmic Genetics & Visual Function Branch, National Eye Institute, National Institutes of Health, Bethesda, Maryland, USA;; b Department of Neurology, Children’s National Medical Center, Washington, District of Columbia, USA

**Keywords:** Kearns-Sayre syndrome, retinoschisis, mitochondria

## Abstract

**Background:**

Kearns-Sayre Syndrome (KSS) is characterized by pigmentary retinopathy, external ophthalmoplegia and heart block. We report on a now 24-year-old male with clinical retinoschisis and molecularly confirmed KSS.

**Materials and Methods:**

Physical and complete ophthalmic examination, molecular diagnosis.

**Results:**

Over nine years of follow-up, the subject manifested progressive signs and symptoms of KSS, including external ophthalmoplegia/strabismus, ptosis, pigmentary retinopathy, corneal edema, Type I diabetes mellitus, gut dysmotility, sensorineural deafness and heart block. At age 21 he was incidentally found to have retinoschisis on optical coherence tomography that remained stable over three years follow-up. Sequencing of the *RS1* gene revealed no pathogenic variants, effectively ruling out co-existing X-linked retinoschisis.

**Conclusions:**

These findings suggest retinoschisis may be a rare manifestation of KSS. A trial of a carbonic anhydrase inhibitor was frustrated by coexisting corneal edema associated with the condition.

## Introduction

Kearns-Sayre Syndrome (KSS) is a rare mitochondrial myopathy classically characterized by external ophthalmoplegia, retinitis pigmentosa, and cardiomyopathy/cardiac conduction defects (1,2). Patients usually present before twenty years of age and can exhibit additional signs/symptoms, including: ptosis, cerebellar ataxia, Pearson’s syndrome (sideroblastic anemia, exocrine pancreas dysfunction, diabetes mellitus) (3), auditory/vestibular dysfunction (4), elevated cerebrospinal fluid protein, and chronic diarrhea with villous atrophy (5). KSS is most often due to mitochondrial genome rearrangements, including deletions and/or insertions (3,6,7). Here, we briefly report on a now 24-year-old male with molecularly confirmed KSS and a typical clinical presentation that was accompanied by retinoschisis incidentally discovered on optic coherence tomography.

## Patient and methods

After obtaining written consent, the patient was examined in the Ophthalmic Genetics Clinic at the National Eye Institute under an IRB-approved research protocol that included permission to publish deidentified findings. Evaluations included specular microscopy, color fundus photography, optical coherence tomography (OCT), and full field electroretinogram (ffERG). Mitochondrial genome sequencing was obtained commercially. Sanger sequencing of the exons and exon-intron boundaries of the *RS1* gene were performed after amplification by the polymerase chain reaction. Primers and conditions are available on request. Laboratory work complied with all applicable health and safety regulations.

## Case report/results

The patient presented at age 14 for evaluation of decreased best-corrected visual acuity. He had been diagnosed with KSS based on clinical presentation and a positive molecular diagnosis at age 11. Mitochondrial DNA sequencing identified a large (7.44kb) deletion between np(8637–8648):(16073–16084) with a 12 base pair repeat at the deletion flanking region. The mutation load was approximately 35% heteroplasmy in peripheral blood.

The patient noted that acuity was worse upon waking and seemed to improve somewhat as the day progressed. Vision in dimly lit conditions was also particularly difficult. Past medical history was remarkable for diabetes mellitus, type 1; a dilated aortic root on echocardiogram; and bilateral sensorineural hearing loss treated with sequential cochlear implants. Past ocular history was remarkable for bilateral, asymmetric and variable ptosis, as well as intermittent binocular, mostly vertical diplopia. Both diplopia and ptosis worsened with fatigue. Medications included atenolol for aortic root dilation, coenzyme Q, and carnitine. Family history was negative for individuals with similar symptoms or early-onset vision loss. He denied alcohol or tobacco use.

At initial presentation, best-corrected visual acuity was 20/70. Pupils were round, moderately reactive and without afferent defect. External examination revealed ptosis with a margin-to-reflex distance of 1 mm. Motility was full. Alternate cover test demonstrated a 10PD right hypertropia and 4PD esotropia. Ishihara color plate testing was normal. Slit lamp examination was remarkable for slightly hazy corneas. Dilated fundus exam showed pigment granularity in the macula and mid-periphery with slightly narrowed arterioles and a pink optic nerve. The patient was empirically started on hypertonic saline drops and ointment for corneal edema and asked to return for follow-up and further testing.

On follow-up seven months later, the patient was subjectively helped with hypertonic drops/ointment and continued to note intermittent diplopia and variable ptosis. Best corrected acuity was 20/40 OD and 20/63 OS. Central corneal thicknesses were 816 μm OD and 832 μm OS with endothelial cell counts of 1408/mm^2^ and 1757/mm^2^, respectively. Goldmann visual fields were constricted to the I1e isopter, but preserved to the V4e and borderline to the I4e isopters. The remainder of the exam was unchanged. Full-field electroretinography demonstrated normal scotopic but mildly reduced photopic responses, consistent with an early cone-rod degeneration. Cirrus OCT qualitatively showed early thinning of the outer nuclear layer, with no clear schisis cavities.

**Figure 1.**
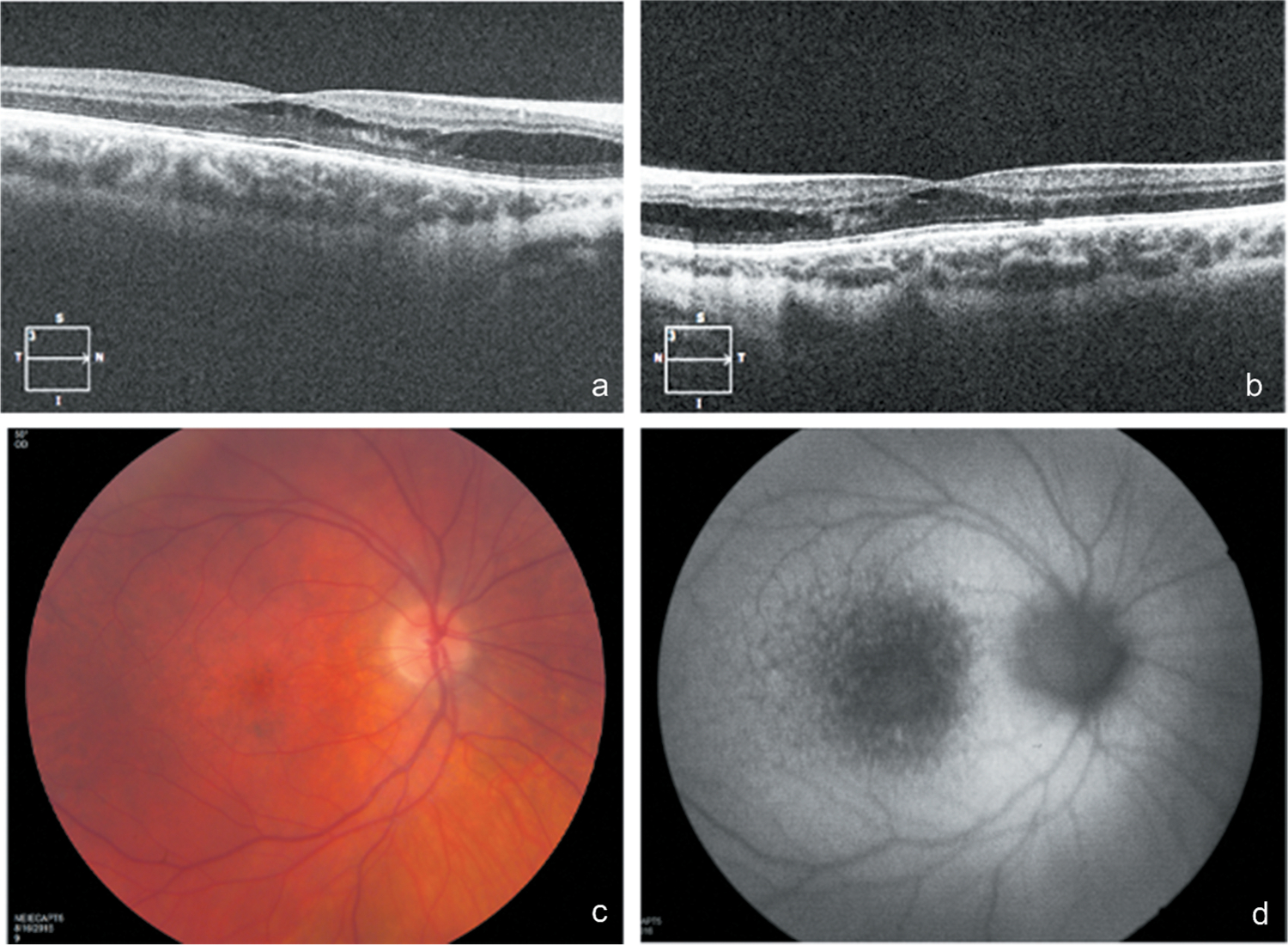
Spectral-domain OCT image of the retina through the fovea of the right (a) and left (b) eyes demonstrating retinoschisis. To highlight details of the schisis cavity, the contrast was increased from the original images. Fundoscopy demonstrated mild pigment mottling of the macula (c), better appreciated as hypoautofluorecent stippling on blue light autofluorescence (d).

The patient continued to be followed on an approximately yearly basis for several years. By age 19, his scotopic ffERG had become mildly decreased, accompanied by continued worsening of photopic signals (mild to moderate). Bright flash responses were not electronegative. A corneal specialist consultation found he was not a good candidate for surgical intervention. At age 21, he was incidentally noted to have developed bilateral retinoschisis on Cirrus OCT, with schisis mostly in the outer plexiform layer (Figure 1a). Acuity was reduced to 20/63 OD and 20/125 OS, which was felt to be a real, significant change at the time. Although the cause was judged to be likely secondary to corneal edema, a contribution from schisis could not be excluded. Refraction at that time was −6.75 + 4.00×120 OD and −8.75 + 4.75×55 length data are not available, but neither fundoscopy nor OCT suggested a posterior staphyloma or other signs of myopic maculopathy. Although use of carbonic anhydrase inhibitor therapy was considered, it was not pursued due to continued corneal edema. Scans on subsequent follow-up over the past two years have shown approximately stable splitting. Although acuity remained stable in the left eye, it continued to drop in the right, last measuring 20/100 at age 23. In addition, mottling of blue-light autofluorescence signal was noted, particularly in the macula, consistent with his cone-rod degeneration (Figure 1b).

Sequencing of the coding regions and intron-exon boundaries of the *RS1* gene, which causes X-linked retinoschisis (8), demonstrated no pathogenic variants, effectively ruling out this condition, as this test has >99% analytic sensitivity of diagnosing this condition.

## Discussion

Mutations in the mitochondrial genome are well-known causes of ophthalmic disease, including Leber hereditary optic atrophy (LHON) and chronic progressive ophthalmoplegia (CPEO), as well as KSS (2). While the precise reasons for this range of phenotypes are not clear, impairment in mitochondrial oxidative phosphorylation is likely a shared mechanism. The variable ptosis, ophthalmoplegia, and photoreceptor dysfunction are classic for KSS. Corneal edema, presumed to be due to mitochondrial dysfunction in the endothelium, is a less common, but recognized manifestation (9,10). To our knowledge, retinoschisis is a novel finding in KSS.

Retinoschisis in young males that centers in the macula is most often associated with X-linked juvenile retinoschisis (XLRS) (11). XLRS (OMIM#312700) is caused by mutations in the *RS1* gene (OMIMN*30089) on Xp22 that codes for the RETINOSCHISIN protein (RS1 protein) (8). As molecular diagnostic testing nearly always identifies a pathogenic variant, normal *RS1* sequence coupled with the absence of an electronegative ERG essentially rules out this diagnosis. Myopic foveal retinoschisis, stellate nonhereditary idiopathic foveomacular retinoschisis (SNIFR), and autosomal recessive familial foveal retinoschisis should also be included on the differential diagnosis (12–14). However, most patients with myopic foveal retinoschisis have higher degrees of myopia than our patient and/or evidence of posterior staphyloma. SNIFR is–as its name implies – a diagnosis of exclusion that has also never been associated with KSS. Autosomal recessive familial foveal retinoschisis due to biallelic *CRB1* mutations generally displays a more spoke-wheel-like pattern, with better visual acuity and preserved ffERG. Lastly, advanced cystic degeneration reminiscent of retinoschisis is sometimes a characteristic of retinal dystrophies such as enhanced S-cone syndrome (15) and it is possible that a similar process is occurring in our patient, with “cysts” large and confluent enough to be reminiscent of retinoschisis.

The pathogenesis of retinoschisis in our KSS patient is unclear, but we can venture some hypotheses based on our current understanding of the RS1 protein’s function. The RS1 protein is a secreted retinal glycoprotein that may help maintain cell-to-cell adhesions, particularly at the photoreceptor-bipolar cell synapse (16,17). RS1 protein physically interacts with subunits of the sodium-potassium ATPase (Na/K ATPase) isoforms found in the retina that affect both metabolic functions such as osmolarity and, importantly, cell-cell adhesions (18,19). As its name implies, the Na/K ATPase requires ATP for its proper function and it is plausible that changes in cellular energetics resulting from mitochondrial dysfunction could, indeed, lead to its dysfunction; in this case, the retinoschisis would be an indirect effect of reduced ATPase function. Regardless of the mechanism, the absence of an electronegative ffERG rules out a retina-wide disturbance of the photoreceptor-bipolar cell synapse.
